# Infantile Scurvy

**Published:** 1907-05-25

**Authors:** 


### Diseases of Children.

### INFANTILE SCURVY.

Many cases of scurvy are overlooked in infancy because the symptoms are slight or the affection is unsuspected, yet the disease is by no means uncommon and the results of proper treatment are quickly obtained, and most gratifying to all concerned. The affection has been described under the names of acute, hæmorrhagic, and scurvy rickets. It is time to give up all these names, and to realise that there is absolutely no connection with rickets, except in so far as the diet which produces infantile scurvy may also cause rickets coincidently.

It is almost invariably in hand-fed infants that it occurs, as the result of a prolonged diet of cooked milk, whether sterilised, pasteurised, or boiled, of condensed milk, or of proprietary foods. Thus it must be ascribed to the lack of the fresh element, or to some particular constituent in uncooked food. In the few breast-fed infants in which it has been reported, the milk was of poor quality, deficient in fat and proteid, and perhaps contained some deleterious element. It has been ascribed to alterations in or deficiency of citric acid in the food; to a kind of acid-intoxication; and to the ptomaines of tainted animal food. The latter theory will certainly not apply to those infants fed on proprietary foods which contain no animal constituents.

It occurs in either sex and at any period of the year. Climate exerts no influence, except that plenty of fresh air and good hygiene may retard its appearance. It is more common among the better classes than among hospital patients, for the children of the former are frequently fed on a rigidly restricted diet of some form of cooked milk or proprietary food, whereas the infants of the poor often get tastes of various antiscorbutic foods at the parents' meals. It is important to realise that it is the prolonged use of these foods which induces scurvy. Thus an infant developed it in a mild form after taking a diet of cream, milk, sugar, and water, carefully sterilised for 20 minutes, for a period of eight months; and another one had a more severe attack after taking pasteurised milk for a year.

The majority of those attacked are from 6 to 12 months of age. Few are under 6 or over 15 months. In breast-fed infants cases have been noted at the age of three weeks, one month, and six weeks.

### Symptoms and Course.

The infants are usually fat and pale. Gradually they fail in health, are fretful, lose weight, lose appetite, and cry when moved or even when approached, for fear of being moved. The onset is gradual, and possibly the immobility of a limb is the first sign to attract attention. Sometimes there is a history of a previous attack of the same nature, lasting for a few days only. If so, it will be found that on account of the child's health some temporary change had been made in the diet, a change which was antiscorbutic in character. In mild cases there may be nothing but anæmia, irritability, and crying on movement; or slight bleeding from a mucous membrane; or some duskiness of the gums. The gums afford the most valuable aid to diagnosis, provided some of the teeth are cut. Typically they are swollen, hyperæmic, varying in colour from a dusky to a deep purple, and even ecchymotic, bulbous, ulcerating and bleeding freely. They may be so swollen as to present a fungating mass which conceals the teeth and projects between them.

The limbs present one or more painful, colourless, tender swellings, often symmetrical. The most common situation is the lower third of the thigh, and the least common is the upper limb. The swelling is due to subperiosteal effusion of blood. The epiphysis may be separated by hæmorrhage, and there may be bleeding into the joint. Bleeding may take place from the ear, into the eyelid, behind the eyeball, from any mucous surface, subcutem, and occasionally into the various serous cavities and the cranial cavity. Muscular pains are severe, and cause loss of rest. Fainting attacks are not uncommon. Often there is a moderate degree of fever.

It is extraordinary what errors of diagnosis are made in these cases. Heubner states that he prevented a surgeon amputating a limb for supposed sarcoma. Usually the infant is said to have got rheumatism—a very rare disease at such an early age. The pseudo-paralysis has been mistaken for infantile paralysis, and at first such a mistake is excusable if there is no swelling and the teeth are not cut, for sometimes acute anterior poliomyelitis is accompanied by considerable pain at the onset. The pain is often ascribed to rickets, but it is doubtful whether simple rickets is ever painful. If the swelling is limited to one joint or limb, it may be diagnosed as acute epiphysitis or acute bone disease. Multiple osteomyelitis and syphilitic epiphysitis have also been diagnosed. The latter disease generally occurs in wasted infants under six months of age. Epistaxis may be thought of no account, and renal hæmaturia may be ascribed to sarcoma. Subcutaneous hæmorrhages suggest purpura, and the gums acute leukæmia. Occasionally there is an acute nephritis. Suspect the presence of the disease in every fat, anæmic child, which is said to have pain in the limbs; inquire into the mode of feeding, and examine the gums.

### Treatment.

Treatment may fail in advanced cases by reason of the anæmia and cachexia. In mild cases there will be improvement in a day or two. Put the child on a diet of uncooked milk and fruit juice, if it is under six months of age. Over that age, a little well boiled sieved potato can be added to the milk. In mild cases, if it is unsafe to use fresh milk, give fruit juice and milk just brought to a boil. Sodium lactate may be given, and can do no harm. Iron, arsenic, and cod-liver oil are useful in convalescence, but it is important not to run any risk of upsetting the digestion in the acute stage.

